# **Spin and valley dependent transport and tunneling magnetoresistance in irradiated ferromagnetic WSe**_**2**_ **double barrier junctions**

**DOI:** 10.1038/s41598-024-81964-0

**Published:** 2025-01-06

**Authors:** Ming Li, Zheng-Yin Zhao, Jia-Yi Sheng

**Affiliations:** College of Science, Xuchang University, Xuchang, 461000 China

**Keywords:** Transfer matrix method, WSe_2_, Spin/valley polarization, Landauer-Büttiker formula, Circularly polarized light, TMR, Applied physics, Condensed-matter physics

## Abstract

Spin and valley polarizations (*P*_*s*_ and *P*_*KK’*_) and tunneling magnetoresistance (TMR) are demonstrated in the ferromagnetic/barrier/normal/barrier/ferromagnetic WSe_2_ junction, with the gate voltage and off-resonant circularly polarized light (CPL) applied to the two barrier regions. The minimum incident energy of non-zero spin- and valley-resolved conductance has been derived, which is consistent with numerical calculations and depends on the electric potential *U*, CPL intensity ΔΩ, exchange field* h*, and magnetization configuration: parallel (P) or antiparallel (AP). For the P (AP) configuration, the energy region with *P*_*KK’*_ = -1 or *P*_*s*_ = 1 is wider (narrower) and increases with ΔΩ. As *h* increases, the *P*_*s*_ = 1 (*P*_*KK’*_ = -1 or *P*_*s*_ = 1) plateau becomes wider (narrower) for the P (AP) configuration. As *U* increases, the energy region with *P*_*KK’*_ = -1 increases first and then moves parallel to the *E*_*F*_-axis, and the energy region with *P*_*s*_ = 1 for the P configuration remains unchanged first and then decreases. The energy region for TMR = 1 increases rapidly with *h*, remains unchanged first and then decreases as *U* increases, and has little dependence on ΔΩ. When the helicity of the CPL reverses, the valley polarization will switch. This work sheds light on the design of spin-valley and TMR devices based on ferromagnetic WSe_2_ double-barrier junctions.

## Introduction

In recent years, monolayer transition-metal dichalcogenides (TMDCs) have attracted extensive attention for promising applications in future devices^1–4^. In TMDCs, according to the requirement of time-reversal symmetry^6–9^, the conduction and valence band extrema occur at the two degenerate valleys (*K*, *K*’) located at the corners of the hexagonal Brillouin zone^9^, which can be broken by the helicity of light^10^. In monolayer TMDCs, because of the broken inversion symmetry and the strong spin–orbit coupling (SOC)^6^, the spin and valley degrees of freedom are coupled (spin-valley locking), and the spin splitting of the valence band is opposite at the two valleys due to the time-reversal symmetry^11^. Therefore, in TMDCs, full spin and valley polarizations can be achieved, which is crucial for spintronics and valleytronics. Significantly, high-quality WSe_2_ with strong SOC exhibits novel behaviors that differ from other TMDCs and two-dimensional materials. Moreover, a monolayer WSe_2_ provides a suitable platform for developing novel spintronics and valleytronics devices, as it is a direct band-gap semiconductor (band gap *E*_g_ = 0.85 eV)^12^.

Several methods have been adopted to manipulate spin and valley degrees of freedom in TMDCs. First is the peculiar magnetic field^13^. The Zeeman effect induced by the magnetic field opens different spin-dependent band gaps at the *K* and *K’* valleys, leading to spin- and valley-polarized transport in normal*/*ferromagnetic*/*normal (N/F/N) WSe_2_ junctions^9^. Second is the magnetic modulation induced through magnetic doping^14^ or proximity effect^15–20^. The magnetic proximity effect in WSe_2_*/*EuS can lead to a giant valley splitting in monolayer WSe_2_^21^. Thirdly, utilizing the optical Stark effect, the pseudomagnetic field induced by the off-resonant CPL is used to select one of the two inequivalent valleys^22–27^. The influence of the off-resonant CPL on the valley polarization in monolayer TMDCs has attracted widespread attention recently^5,12,23–25,28–30^. For example, Hao et al. predicted the quantum spin and valley Hall effects in MoS_2_ irradiated with the off-resonant CPL^12^, and Qiu et al. demonstrated that the perfect spin and valley polarizations in WSe_2_ are caused by the off-resonant CPL and the massive SOC^28^.

Tunneling magnetoresistance (TMR) is another key topic in spintronics, which has been widely applied to storage and magnetic sensor technologies^31^, magnetic random access memory^32^, and hard disk drives^33^. So far, the spin- and valley-dependent transport and TMR have been investigated theoretically and experimentally in many ferromagnetic junctions based on graphene^34,35^, silicene^16,17^, and MoS_2_^18,19,36,37^. Moreover, the spin-valley current in many ferromagnetic junctions based on silicene^38^, MoS_2_^37^, and WSe_2_^4,39,40^ can also be controlled by the off-resonant CPL. Liu et al. demonstrated that the normal/barrier/normal/barrier/normal (N/B/N/B/N) WSe_2_ junction modulated by the off-resonant CPL and gate voltage can function as a valley filter and valley valve device^22^. Hajati et al. found highly spin- and valley-polarized current and high TMR in the ferromagnetic/ferromagnetic/normal (F/F/N) WSe_2_ junction in the presence of gate voltage and off-resonance CPL in the middle ferromagnetic region^40^. However, by designing appropriate geometric structures, it is possible to further improve or modulate the energy region of full spin and valley polarizations as well as large TMR in WSe_2_ junctions. As an extension of the system and model studied in Ref.^22^, here we consider the ferromagnetic/barrier/normal/barrier/ferromagnetic (F/B/N/B/F) WSe_2_ junction, where the same gate voltage and off-resonance CPL are applied in the two barrier regions. The spin- and valley-resolved effective potential for electrons in each region of the F/B/N/B/F WSe_2_ junction will depend on the spin and valley degrees of freedom, the helicity of the off-resonance light, and magnetization configuration. This will certainly affect the spin- and valley-resolved conductance, spin and valley polarizations, and TMR in this junction. Furthermore, to my knowledge, existing literature has not considered the tunneling properties of the F/B/N/B/F WSe_2_ junction, as well as how the spin and valley polarizations and TMR in the junction depend on the exchange field, the electrostatic potential, and the strength of the off-resonance CPL.

This paper discovers full spin and valley polarizations, as well as large TMR in the F/B/N/B/F WSe_2_ junction, with gate voltage and off-resonance CPL applied to the barrier regions. We demonstrate that the energy regions of full spin and valley polarizations and large TMR can be regulated by the electric potential (*U*), CPL intensity (ΔΩ), and exchange field (*h*), and discover the underlying physical mechanisms, which have not been reported in similar ferromagnetic-TMDC junctions^18^. We derived the minimum incident energy for non-zero spin- and valley-resolved conductance, and verified it through numerical calculations. The energy region for TMR = 1 increases (decreases) with *h* (*U*) and has little dependence on ΔΩ. For the P (AP) configuration, the energy region with *P*_*KK’*_ = -1 or *P*_*s*_ = 1 is relatively wider (narrower) and widens as ΔΩ increases. As *h* increases, the energy region with *P*_*s*_ = 1 (*P*_*KK’*_ = -1 or *P*_*s*_ = 1) widens (narrows) for the P (AP) configuration. As *U* increases, the energy region with *P*_*KK’*_ = -1 increases first and then moves parallel to the *E*_*F*_-axis, and the energy region with *P*_*s*_ = 1 for the P configuration remains unchanged first and then decreases. When the helicity of the off-resonance light reverses, the valley polarization will switch, while the spin polarization and TMR will not.

The remainder of the paper is organized as follows. In Sec. II, the theoretical model and the schematic structure of the F/B/N/B/F WSe_2_ junction are presented. Sec. III studies the spin and valley polarizations along with TMR of the WSe_2_ junction in the presence of off-resonant CPL and gate voltage. Finally, Sec. IV gives a summary.

## Theory and model

**Fig. 1:**
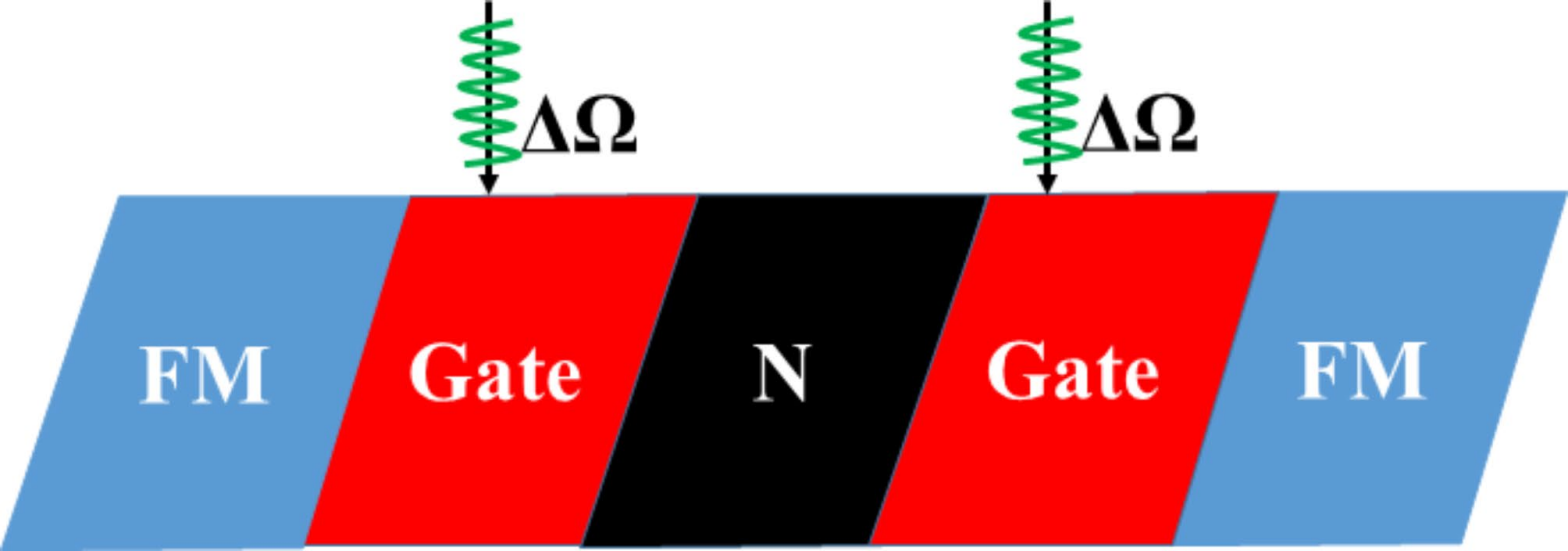
Schematic diagram of WSe_2_-based F/B/N/B/F junction. The two barrier regions are formed by the top gate voltage and illuminated by the off-resonant circularly polarized light.

The proposed symmetric F/B/N/B/F WSe_2_ junction is shown schematically in Fig. 1, where the two barrier regions are formed by the electrostatic potential *U* induced by the gate voltage and illuminated by the off-resonant CPL with a frequency of Ω. An electromagnetic potential can describe the CPL as ***A***(*t*) = [*E*_0_sin(± Ω*t*)/Ω, *E*_0_cos (± Ω*t*)/Ω], where + (-) corresponds to the right-handed (left-handed) circular polarization, and *E*_0_ is the amplitude of the electric field. Due to broken inversion symmetry, monolayer TMDCs exhibit valley-dependent optical interband excitation, i.e. electrons from different valleys are selectively excited by CPLs with different helicities^41^. Moreover, the magnetization orientation in the left ferromagnetic region is assumed to be always positive, while that in the right ferromagnetic region can be positive or negative, resulting in two types of magnetization configurations: parallel (P) and antiparallel (AP).

For *eAv*_*f*_ /Ω <  < 1 (*v*_*f*_ = 5 × 10^5^ m/s is the Fermi velocity in WSe_2_), the low-energy effective Hamiltonian for the proposed WSe_2_ junction is given by^6,22,28,42^:

1 $$H = \hbar v_{F} (k_{x} \tau_{z} \sigma_{x} + k_{y} \sigma_{y} ) + (E_{g} + \tau_{z} \Delta \Omega )\sigma_{z} + \tau_{z} s_{z} (\lambda_{c} \sigma_{ + } + \lambda_{v} \sigma_{ - } ) + U(x) - s_{z} h(x)$$

Here *E*_g_ is the band gap of WSe_2_^22,39^, ΔΩ = (*eAv*_*f*_)^2^/*h*Ω is the effective energy term describing the CPL intensity^43^. *s*_*z*_ =  + 1 (-1) denotes the up (down) spin of electrons, *τ*_*z*_ =  + 1 (-1) stands for the *K* (*K’*) valley. *σ*_*x,y,z*_ represents the Pauli matrix in the sublattice space, and *σ*_±_ = *σ*_0_ ± *σ*_*z*_, with *σ*_0_ being the unit matrix^22,39^. *λ*_*c*_ = 7.5 meV (*λ*_*v*_ = 112.5 meV)^22,39^ is the spin splitting at the conduction (valence) band edge caused by the intrinsic SOC. The last term in Eq. (1) represents the magnetic modulation in the ferromagnetic region, where *h* is the exchange field.

The electrostatic gate potential and off-resonance CPL in the two barrier regions can be defined as $U(x) = U\Theta (x)\Theta (L_{B} - x) + U\Theta (x - L_{B} - L_{W} )\Theta (2L_{B} + L_{W} - x)$ and $\Delta \Omega (x) = \Delta \Omega \Theta (x)\Theta (L_{B} - x) + \Delta \Omega \Theta (x - L_{B} - L_{W} )\Theta (2L_{B} + L_{W} - x)$, respectively, with [$\Theta (x)$] being the Heaviside step function. Moreover, the exchange field in the two ferromagnetic regions can be described as $h(x) = h\Theta ( - x) \pm h\Theta (x - 2L_{B} - L_{W} )$, where + (-) corresponds to the P (AP) magnetization configuration.

The energy dispersion relation in the modulated regions is^22,39^:

2 $$E_{ \pm } = \pm \sqrt {(\hbar v_{F} k)^{2} + (E_{g} + \tau_{z} \Delta \Omega + \tau_{z} s_{z} \lambda_{ - } )^{2} } + \tau_{z} s_{z} \lambda_{ + } + U(x) - s_{z} h(x)$$

with *λ*_±_ = *λ*_*c*_ ± *λ*_*v*_. Thus the spin- and valley-resolved conduction band minimum (CBM) energy of the WSe_2_ junction can be written as:

3 $$E_{{\tau_{z} s_{z} }} = E_{g} + 2\tau_{z} s_{z} \lambda_{c} + \tau_{z} \Delta \Omega (x) + U(x) - s_{z} h(x)$$

**Fig. 2:**
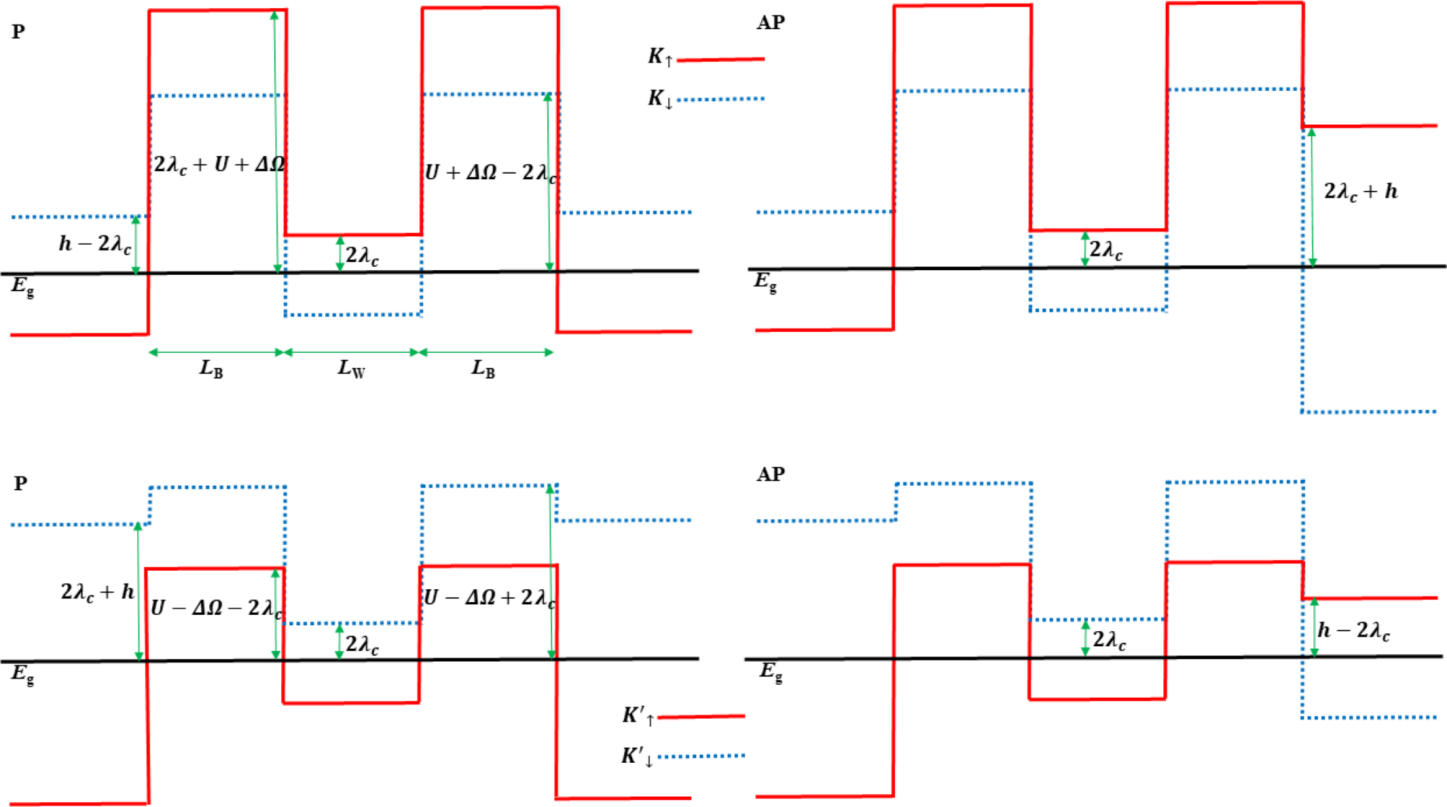
Spin- and valley-resolved conduction band minimum (CBM) energy in each region of the F/B/N/B/F WSe_2_ junction. The width of the two barrier layers is *L*_B_ = 5 nm, and the width of the central well layer is *L*_w_ = 5 nm. The left (right) column corresponds to the P (AP) configuration. The horizontal line denotes the energy level with *E* = *E*_g_ = 850 meV. ↑ (↓) denotes the up (down) spin of electrons, respectively.

As shown in Eqs. (2) and (3), the exchange field, off-resonance CPL, and SOC collectively lift the spin and valley degeneracy of the energy dispersion relation. Figure 2 shows the spin- and valley-resolved CBM energy (effective potential for electrons) in each region of the F/B/N/B/F WSe_2_ junction, which depends on the spin and valley degrees of freedom and magnetization configuration, and will affect the spin- and valley-resolved conductance, spin and valley polarizations, as well as TMR of this junction. As shown in Eqs. (2) and (3), $E_{{Ks_{z} }}$ ($E_{{K^{\prime}s_{z} }}$) for ΔΩ > 0 approximately equals $E_{{K^{\prime}s_{z} }}$ ($E_{{Ks_{z} }}$) for ΔΩ < 0, because the term $\left| {2\tau_{z} s_{z} \lambda_{c} } \right|$= 15 meV is relatively small. Therefore, when ΔΩ reverses the sign, the valley polarization (*P*_*KK’*_) will reverse the sign synchronously, while the spin polarization (*Ps*) and TMR will not be deeply affected. Thus, we will only discuss the case where ΔΩ > 0.

As can be concluded from Eq. (1) and Fig. 2, the critical minimum incident energy of non-zero spin- and valley-resolved conductance ($E_{{c\tau_{z} s_{z} }}$) equals the highest CBM energy of the F/B/N/B/F WSe_2_ junction, which can be expressed as follows:

4 $$E_{{cK \uparrow }} = \left\{ {\begin{array}{*{20}l} {E_{g} + 2\lambda _{c} + U + \Delta \Omega } \hfill & {{\text{for}}\;{\text{P}}\;{\text{configuration,}}} \hfill \\ {E_{g} + 2\lambda _{c} + \max \{ h,U + \Delta \Omega \} } \hfill & {{\text{for}}\;{\text{AP}}\;{\text{configuration,}}} \hfill \\ \end{array} } \right.$$

5 $$E_{{cK \downarrow }} = E_{g} - 2\lambda _{c} + \max \{ h,U + \Delta \Omega \} \quad {\text{for}}\;{\text{both}}\;{\text{configurations,}}$$

6 $$E_{{cK' \uparrow }} = \left\{ {\begin{array}{*{20}l} {E_{g} - 2\lambda _{c} + \max \{ 0,U - \Delta \Omega \} } \hfill & {{\text{for}}\;{\text{P}}\;{\text{configuration,}}} \hfill \\ {E_{g} - 2\lambda _{c} + \max \{ h,U - \Delta \Omega \} } \hfill & {{\text{for}}\;{\text{AP}}\;{\text{configuration,}}} \hfill \\ \end{array} } \right.$$

7 $$E_{{cK' \downarrow }} = E_{g} + 2\lambda _{c} + \max \{ h,U - \Delta \Omega \} \;{\text{for}}\;{\text{both}}\;{\text{configurations}}.$$

The wave function in each region can be expressed in the following form^22^:

8 $$\Phi (x) = a\left( {\begin{array}{*{20}c} 1 \\ {\frac{{\hbar v_{F} k_{ + } }}{\delta }} \\ \end{array} } \right)e^{iqx} + b\left( {\begin{array}{*{20}c} 1 \\ {\frac{{\hbar v_{F} k_{ - } }}{\delta }} \\ \end{array} } \right)e^{ - iqx}$$

Here $\delta = E - U + s_{z} h(x) - 2\tau_{z} s_{z} \lambda_{v} + E_{g} + \tau_{z} \Delta \Omega$, *a* and *b* are the scattering coefficients. The parallel and perpendicular wave vectors in each region are

$$k_{y} = \frac{{\sqrt {(E - \tau_{z} s_{z} \lambda_{ + } - U + s_{z} h(x))^{2} - (E_{g} + \tau_{z} \Delta \Omega + \tau_{z} s_{z} \lambda_{ - } )^{2} } }}{{\hbar v_{F} }}\sin \theta$$

9 $$q^{2} = \frac{{(E - \tau_{z} s_{z} \lambda_{ + } - U + s_{z} h(x))^{2} - (E_{g} + \tau_{z} \Delta \Omega + \tau_{z} s_{z} \lambda_{ - } )^{2} }}{{(\hbar v_{F} )^{2} }} - k_{{_{y} }}^{2}$$

Here *θ* denotes the incident angle. Using the continuity condition of the wave function at the interfaces and the transfer-matrix method, the spin- and valley-dependent transmission probability ($T_{{\tau_{z} s_{z} }}$) can be calculated. Then, the conductance at zero temperature is given by the Landauer-Büttiker formula^44^:

10 $$G_{{\tau_{z} s_{z} }} = G_{0} \int {T_{{\tau_{z} s_{z} }} } \cos \theta d\theta$$

Here *G*_0_ = 2e^2^*/h* is the quantum conductance.

The spin- and valley-resolved conductance can be written as^45,46^:

11 $$G_{ \uparrow ( \downarrow )} = \left( {G_{K \uparrow ( \downarrow )} + G_{K^{\prime} \uparrow ( \downarrow )} } \right)/2$$

12 $$G_{K(K^{\prime})} = {(}G_{K(K^{\prime}) \uparrow } + G_{K(K^{\prime}) \downarrow } {)}/2$$

Using the spin- and valley-resolved conductance, the valley and spin polarizations (*P*_*KK’*_ and *P*_*s*_) are defined as follows^4^:

13 $$P_{KK^{\prime}} = {{{(}G_{K} - G_{K^{\prime}} {)}} \mathord{\left/ {\vphantom {{{(}G_{K} - G_{K^{\prime}} {)}} {{(}G_{K} + G_{K^{\prime}} {)}}}} \right. \kern-0pt} {{(}G_{K} + G_{K^{\prime}} {)}}}$$

14 $$P_{s} = {{{(}G_{ \uparrow } - G_{ \downarrow } {)}} \mathord{\left/ {\vphantom {{{(}G_{ \uparrow } - G_{ \downarrow } {)}} {{(}G_{ \uparrow } + G_{ \downarrow } {)}}}} \right. \kern-0pt} {{(}G_{ \uparrow } + G_{ \downarrow } {)}}}$$

Finally, TMR can be defined as^39^:

15 $${\text{TMR}} = {{\left( {G_{P} - G_{AP} } \right)} \mathord{\left/ {\vphantom {{\left( {G_{P} - G_{AP} } \right)} {G_{P} }}} \right. \kern-0pt} {G_{P} }}$$

Here *G*_*P*_ (*G*_*AP*_) is the total conductance of the F/B/N/B/F WSe_2_ junction in the P (AP) configuration, with $G_{P(AP)} = G_{ \uparrow } + G_{ \downarrow } = G_{K} + G_{K^{\prime}}$^19^.

## Results and discussion

In this section, we calculate the spin- and valley-dependent conductance and TMR in the F/B/N/B/F WSe_2_ junction for both parallel and antiparallel magnetization configurations in the presence of off-resonant CPL and gate voltage. Firstly, the cases of electric potential *U* = 100 meV, exchange field *h* = 200 meV, and different CPL intensity (ΔΩ) are explored. Secondly, the cases of ΔΩ = 200 meV, *h* = 200 meV, and different *U* are studied. Finally, the cases of *U* = 100 meV, ΔΩ = 200 meV, and various *h* are examined.

**Fig. 3:**
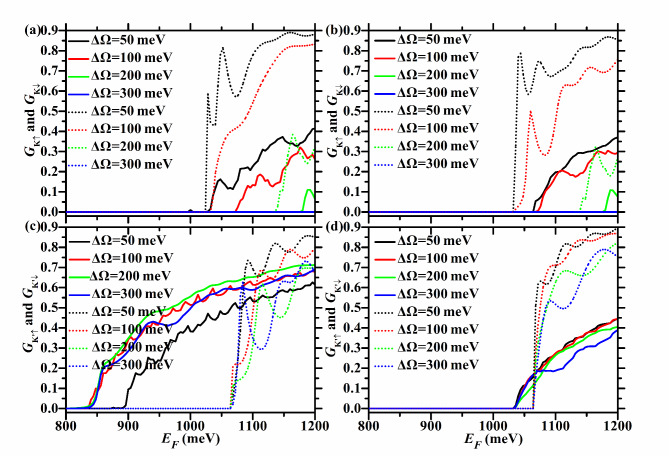
Spin- and valley-resolved conductance of the F/B/N/B/F WSe_2_ junction with ΔΩ = 50, 100, 200, and 300 meV. Here *h* = 200 meV and *U* = 100 meV. In the left (right) column, the junction is in the P (AP) configuration. The solid (dotted) lines correspond to spin-up (-down) electrons.

**Fig. 4:**
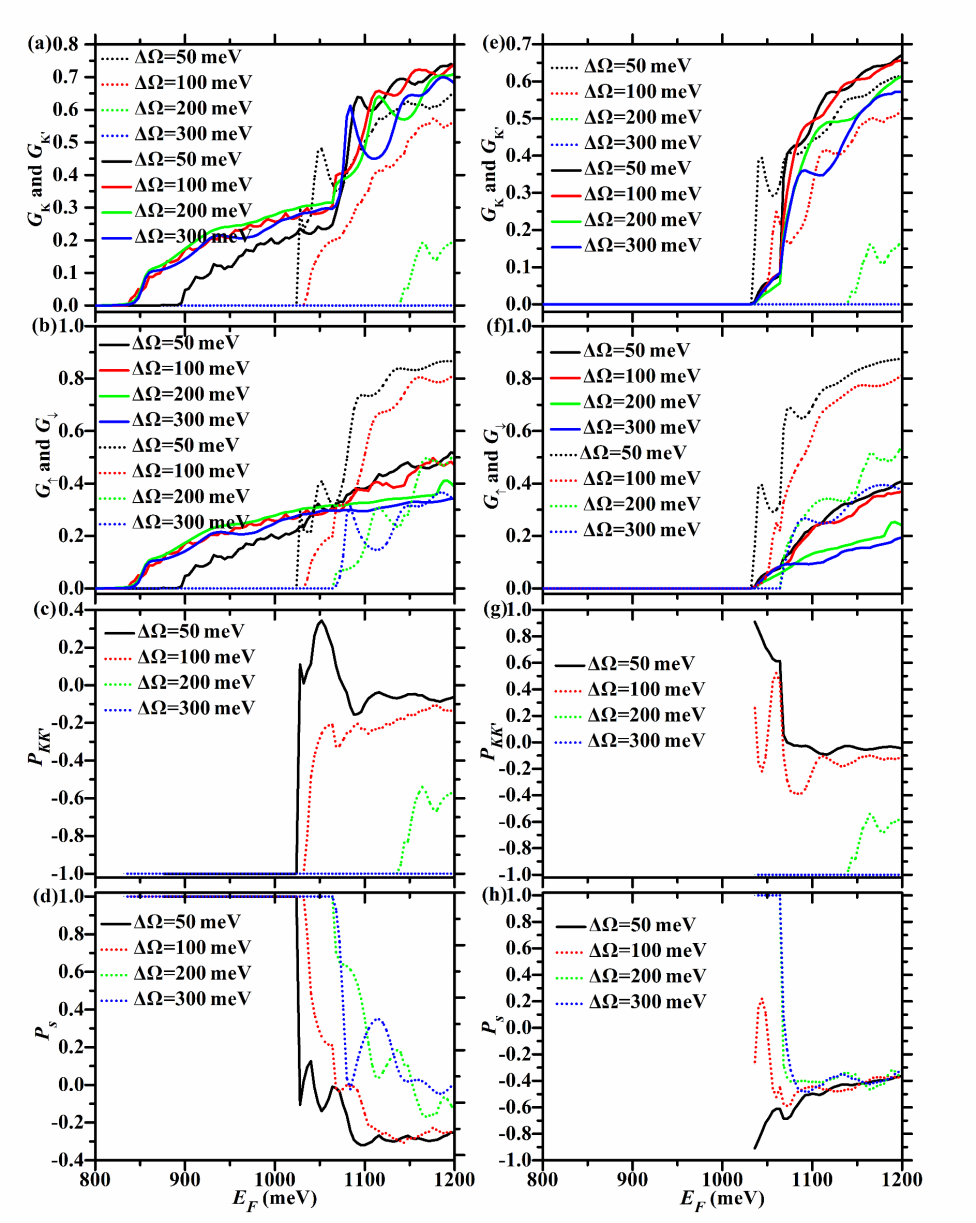
*G*_*K*_ (dotted lines),* G*_*K’*_ (solid lines), *G*_↑_ (solid lines), *G*_↓_ (dotted lines), *P*_*KK’*_, and *P*_*s*_ versus *E*_*F*_ for the F/B/N/B/F WSe_2_ junction with ΔΩ = 50, 100, 200, and 300 meV. Here *h* = 200 meV and *U* = 100 meV. In the left (right) column, the junction is in the P (AP) configuration.

Figures 3 and 4 show the spin- and valley-resolved conductance of the F/B/N/B/F WSe_2_ junction in P and AP configurations with *U* = 100 meV, *h* = 200 meV, and different ΔΩ. Figure 9 exhibits the corresponding total conductance as well as TMR, and Tables 1 and 2 depict the corresponding critical incident energy for the non-zero spin- and valley-resolved conductance.

**Table 1:** The critical incident energy (in units of meV) of non-zero spin- and valley-resolved conductance of the F/B/N/B/F WSe_2_ junction in the P configuration with *U* = 100 meV, *h* = 200 meV, and ΔΩ = 50, 100, 200, and 300 meV, respectively.

ΔΩ	*E*_*cK*_$_\uparrow$	*E*_*cK*_$_\downarrow$	*E*_*cK’*_$_\uparrow$	*E*_*cK’*_$_\downarrow$	*E*_*cK*_	*E*_*cK’*_	*E*_*c*_$_\uparrow$	*E*_*c*_$_\downarrow$
50	1028	1028	876	1068	1028	876	876	1028
100	1076	1036	836	1068	1036	836	836	1036
200	1180	1140	812	1068	1140	812	812	1068
300	> 1200	> 1200	824	1068	> 1200	824	824	1068

**Table 2:** The critical incident energy (in units of meV) of non-zero spin- and valley-resolved conductance of the F/B/N/B/F WSe_2_ junction in the AP configuration with *U* = 100 meV, *h* = 200 meV, and ΔΩ = 50, 100, 200, and 300 meV, respectively.

ΔΩ	*E*_*cK*_$_\uparrow$	*E*_*cK*_$_\downarrow$	*E*_*cK’*_$_\uparrow$	*E*_*cK’*_$_\downarrow$	*E*_*cK*_	*E*_*cK’*_	*E*_*c*_$_\uparrow$	*E*_*c*_$_\downarrow$
50	1068	1036	1036	1068	1036	1036	1036	1036
100	1068	1036	1036	1068	1036	1036	1036	1036
200	1176	1140	1036	1068	1140	1036	1036	1068
300	> 1200	> 1200	1036	1068	> 1200	1036	1036	1068

For *U* = 100 meV and *h* = 200 meV, $U + \Delta \Omega > 0$ and $U - \Delta \Omega < h$ hold. So $E_{cK {\text{'}}\downarrow } = E_{g} + 2\lambda_{c} + h = 1065$ meV, and it is the same for both P and AP configurations and does not change with ΔΩ, as shown in Fig. 3(c) and (d), as well as Tables 1 and 2. For the P case, $E_{cK \uparrow } = E_{g} + 2\lambda_{c} + U + \Delta \Omega = 965 + \Delta \Omega$ meV, and increases with ΔΩ, as seen in Fig. 3(a) and Table 1. For the AP case, $E_{cK{\text{'}} \uparrow} = E_{g} - 2\lambda_{c} + h = 1035$ meV, and does not change with ΔΩ, as illustrated in Fig. 3(d) and Table 2.

When ΔΩ < 100 meV,$U - \Delta \Omega > 0$ and $U + \Delta \Omega < h$ hold. So $E_{cK \downarrow } = E_{g} - 2\lambda_{c} + h = 1035$ meV, and it is the same for both P and AP configurations and does not vary with ΔΩ, as depicted in Fig. 3(a) and (b), as well as Tables 1 and 2.

For the P case, $E_{cK{\text{'}} \uparrow } = E_{g} - 2\lambda_{c} + U - \Delta \Omega = 935 - \Delta \Omega$ meV, and decreases with ΔΩ, as described in Fig. 3(c) and Table 1. So $E_{cK} = \min \{ E_{cK \uparrow } ,E_{cK \downarrow } \} = \min \{ 965 + \Delta \Omega ,1035\}$ meV, $E_{cK{\text{'}} } = E_{cK{\text{'}} \uparrow } = 935 - \Delta \Omega$ meV, $E_{c \uparrow } = E_{cK{\text{'}} \uparrow } = 935 - \Delta \Omega$ meV, *E*_*c*↓_ = *E*_*cK*↓_ = 1035 meV, as seen in Fig. 4(a) and (b), and Table 1. Therefore, for the P case, *P*_*KK’*_ = -1 in the energy region [$935 - \Delta \Omega$,$\min \{ 965 + \Delta \Omega ,1035\}$ meV], which is relatively wide and increases with ΔΩ, as shown in Fig. 4(c). *P*_*s*_ = 1 in the energy region [$935 - \Delta \Omega$,1035 meV], which is relatively wide and increases with ΔΩ, as shown in Fig. 4(d).

For the AP case, $E_{cK \uparrow } = E_{g} + 2\lambda_{c} + h = 1065$ meV, and does not vary with ΔΩ, as seen in Fig. 3(b) and Table 2. So *E*_*cK*_ = *E*_*cK*↓_ = 1035 meV, *E*_*cK’*_ = *E*_*cK’*↑_ = 1035 meV*, E*_*c*↑_ = *E*_*cK’*↑_ = 1035 meV, *E*_*c*↓_ = *E*_*cK*↓_ = 1035 meV, as seen in Fig. 4(e) and (f), and Table 2. Therefore, for the AP case, the energy region with *P*_*KK’*_ = -1 (*P*_*s*_ = 1) does not exist, as shown in Fig. 4(g), (h).

TMR = 1 in the energy region [$935 - \Delta \Omega$,1035 meV], which is relatively wide and increases with ΔΩ, and its upper limit does not change with ΔΩ, as shown in Fig. 9(a) and (b).

When ΔΩ ≥ 100 meV, $U - \Delta \Omega \le 0$ and $U + \Delta \Omega \ge h$ hold. So $E_{cK \downarrow } = E_{g} - 2\lambda_{c} + U + \Delta \Omega = 935 + \Delta \Omega$ meV, and increases with ΔΩ, regardless of P or AP configuration, as depicted in Fig. 3(a) and (b), as well as Tables 1 and 2.

For the P case, $E_{cK{\text{'}} \uparrow } = E_{g} - 2\lambda_{c} = 835$ meV, and does not vary with ΔΩ, as seen in Fig. 3(c) and Table 1. So $E_{cK} = E_{cK \downarrow } = E_{g} - 2\lambda_{c} + U + \Delta \Omega = 935 + \Delta \Omega$ meV, $E_{cK{\text{'}} } = E_{cK{\text{'}} \uparrow } = E_{g} - 2\lambda_{c} = 835$ meV, $E_{c \uparrow } = E_{cK{\text{'}} \uparrow } = E_{g} - 2\lambda_{c} = 835$ meV, $E_{c \downarrow } = \min \{ 935 + \Delta \Omega ,1065\}$ meV, as seen in Fig. 4(a) and (b) and Table 1. For the P case, *P*_*KK’*_ = -1 in the energy region [$835$,$935 + \Delta \Omega$ meV], which is relatively wide and increases with ΔΩ, as seen in Fig. 4(c). As shown in Fig. 4(d), *P*_*s*_ = 1 in the energy region [835,$\min \{ 935 + \Delta \Omega ,1065\}$ meV]. This increases with ΔΩ when 100 < ΔΩ < 130 meV, and remains at [835,1065 meV] when ΔΩ ≥ 130 meV, with a width of 230 meV.

For the AP case, $E_{cK \uparrow } = E_{g} + 2\lambda_{c} + U + \Delta \Omega = 965 + \Delta \Omega$ meV, and increases with ΔΩ, as seen in Fig. 3(b) and Table 2. So $E_{cK} = E_{cK \downarrow } = 935 + \Delta \Omega$ meV, *E*_*cK’*_ = *E*_*cK’*↑_ = 1035 meV, *E*_*c*↑_ = *E*_*cK’*↑_ = 1035 meV, $E_{{c \downarrow }} = \min \{ 935 + \Delta \Omega ,1065\}$ meV, as seen in Fig. 4(e) and (f) and Table 2. For the AP case, *P*_*KK’*_ = -1 in the energy region [1035,$935 + \Delta \Omega$ meV], which increases with ΔΩ, as shown in Fig. 4(g). As shown in Fig. 4(h), *P*_*s*_ = 1 in the energy region [1035,$\min \{ 935 + \Delta \Omega ,1065\}$ meV]. This region increases with ΔΩ when 100 < ΔΩ < 130 meV, and remains at [1035,1065 meV] when ΔΩ ≥ 130 meV, with a width of 30 meV.

Therefore, when ΔΩ ≥ 100 meV, TMR = 1 in the energy region [835,1035 meV], which is relatively wide and remains at 200 meV, as described in Fig. 9(a) and (b).

**Fig. 5:**
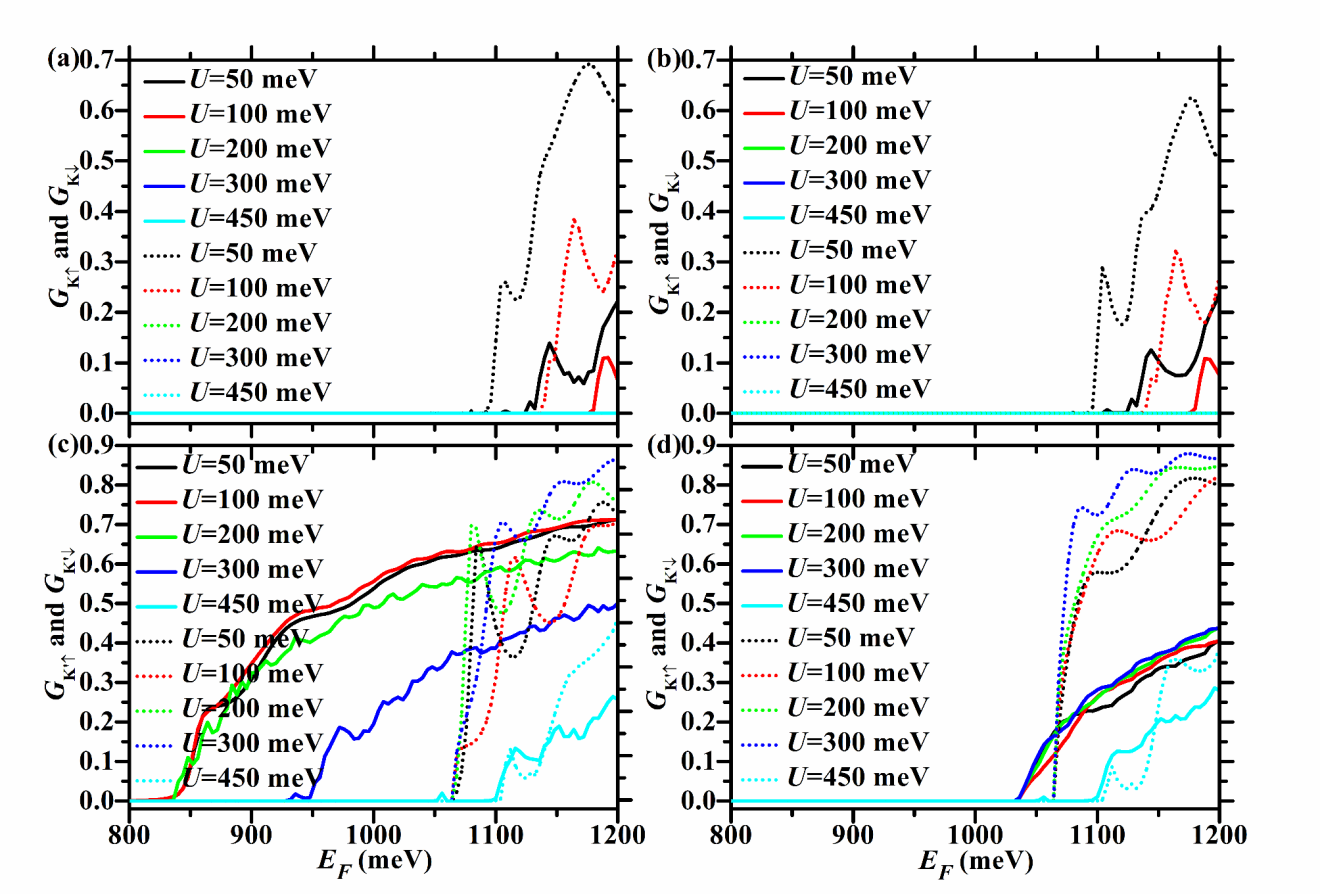
Spin- and valley-resolved conductance of the F/B/N/B/F WSe_2_ junction with ΔΩ = 200 meV, *h* = 200 meV, and *U* = 50, 100, 200, 300 and 450 meV. The solid (dotted) lines correspond to spin-up (-down) electrons. In the left (right) column, the junction is in the P (AP) configuration.

**Fig. 6:**
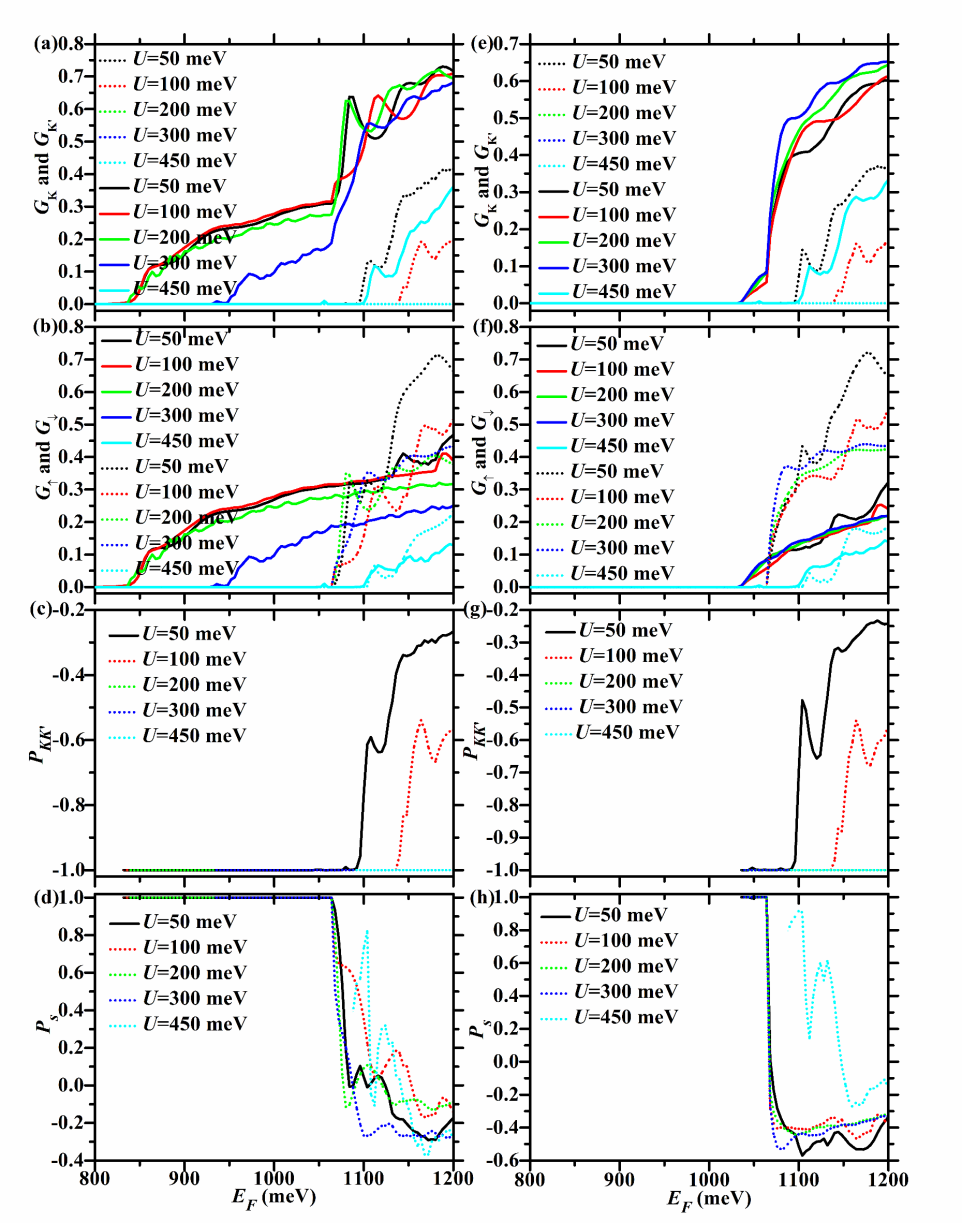
*G*_*K*_ (dotted lines),* G*_*K’*_ (solid lines), *G*_↑_ (solid lines), *G*_↓_ (dotted lines), *P*_*KK’*_, and *P*_*s*_ versus *E*_*F*_ for the F/B/N/B/F WSe_2_ junction with *U* = 50, 100, 200, 300, and 450 meV. Here *h* = 200 meV and ΔΩ = 200 meV. In the left (right) column, the junction is in the P (AP) configuration.

Figures 5 and 6 show the spin- and valley-resolved conductance of the F/B/N/B/F WSe_2_ junction in P and AP configurations with ΔΩ = 200 meV, *h* = 200 meV, and different *U*. Figure 9 exhibits the corresponding total conductance as well as TMR, and Tables 3 and 4 depict the corresponding critical incident energy for the non-zero spin- and valley-resolved conductance.

**Table 3:** The critical incident energy (in units of meV) of non-zero spin- and valley-resolved conductance of the F/B/N/B/F WSe_2_ junction in the P configuration with ΔΩ = 200 meV, *h* = 200 meV, and *U* = 50, 100, 200, 300 and 450 meV, respectively.

*U*	*E*_*cK*_$_\uparrow$	*E*_*cK*_$_\downarrow$	*E*_*cK’*_$_\uparrow$	*E*_*cK’*_$_\downarrow$	*E*_*cK*_	*E*_*cK’*_	*E*_*c*_$_\uparrow$	*E*_*c*_$_\downarrow$
50	1128	1092	820	1068	1092	820	820	1068
100	1180	1140	812	1068	1140	812	812	1068
200	> 1200	> 1200	836	1068	> 1200	836	836	1068
300	> 1200	> 1200	932	1068	> 1200	932	932	1068
450	> 1200	> 1200	1096	1100	> 1200	1096	1096	1100

**Table 4:** The critical incident energy (in units of meV) of non-zero spin- and valley-resolved conductance of the F/B/N/B/F WSe_2_ junction in the AP configuration with ΔΩ = 200 meV, *h* = 200 meV, and *U* = 50, 100, 200, 300 and 450 meV, respectively.

*U*	*E*_*cK*_$_\uparrow$	*E*_*cK*_$_\downarrow$	*E*_*cK’*_$_\uparrow$	*E*_*cK’*_$_\downarrow$	*E*_*cK*_	*E*_*cK’*_	*E*_*c*_$_\uparrow$	*E*_*c*_$_\downarrow$
50	1108	1092	1036	1068	1092	1036	1036	1068
100	1176	1140	1036	1068	1140	1036	1036	1068
200	> 1200	> 1200	1036	1068	> 1200	1036	1036	1068
300	> 1200	> 1200	1036	1068	> 1200	1036	1036	1068
450	> 1200	> 1200	1096	1100	> 1200	1096	1096	1100

For ΔΩ = 200 meV and *h* = 200 meV, $U + \Delta \Omega > h$ holds. So $E_{cK \uparrow } = E_{g} + 2\lambda_{c} + U + \Delta \Omega = 1065 + U$ meV, $E_{cK \downarrow } = E_{g} - 2\lambda_{c} + U + \Delta \Omega = 1035 + U$ meV, and they are the same for both P and AP configurations and increase with *U*, as seen in Fig. 5(a) and (b), as well as Tables 3 and 4.

When* U* < 200 meV, $U - \Delta \Omega < 0 < h$ holds. So $E_{cK{\text{'}} \downarrow } = E_{g} + 2\lambda_{c} + h = 1065$ meV, and does not change with *U*, whether P or AP configuration, as shown in Fig. 5(c) and (d), as well as Tables 3 and 4.

For the P case, $E_{cK{\text{'}} \uparrow } = E_{g} - 2\lambda_{c} = 835$ meV, and does not change with ΔΩ, as seen in Fig. 5(c) and Table 3. So $E_{cK} = E_{cK \downarrow } = 1035 + U$ meV, *E*_*cK’*_ = *E*_*cK’*↑_ = 835 meV, *E*_*c*↑_ = *E*_*cK’*↑_ = 835 meV, $E_{c \downarrow } = \min \{ 1065,1035 + U\}$ meV, as illustrated in Fig. 6(a) and (b) and Table 3. Therefore, for the P case, *P*_*KK’*_ = -1 in the energy region [835,$1035 + U$ meV], which increases with* U*, as seen in Fig. 6(c). As shown in Fig. 6(d), *P*_*s*_ = 1 in the energy region [835,$\min \{ 1065,1035 + U\}$ meV]. This region increases with *U* when 0 < *U* < 30 meV, and remains at [835,1065 meV] when 30 ≤ *U* < 200 meV, with a width of 230 meV.

For the AP case, $E_{cK{\text{'}} \uparrow } = E_{g} - 2\lambda_{c} + h = 1035$ meV, and does not change with *U*, as seen in Fig. 5(d) and Table 4. So $E_{cK} = E_{cK \downarrow } = 1035 + U$ meV, *E*_*cK’*_ = *E*_*cK’*↑_ = 1035 meV*, E*_*c*↑_ = *E*_*cK’*↑_ = 1035 meV, $E_{c \downarrow } = \min \{ 1065,1035 + U\}$ meV, as seen in Fig. 6(e) and (f) and Table 4. For the AP case, *P*_*KK’*_ = -1 in the energy region [1035,$1035 + U$ meV], which increases with *U*, as seen in Fig. 6(g). As shown in Fig. 6(h), *P*_*s*_ = 1 in the energy region [1035,$\min \{ 1065,1035 + U\}$ meV]. This region increases with *U* when 0 < *U* < 30 meV, and remains at [1035,1065 meV] when 30 ≤ *U* < 200 meV, with a width of 30 meV.

Therefore, when* U* < 200 meV, TMR = 1 in the energy region [835,1035 meV], which is relatively wide and remains at 200 meV, as shown in Fig. 9(c) and (d).

When 200 ≤ *U* < 400 meV, $0 < U - \Delta \Omega < h$ holds. So $E_{cK{\text{'}} \downarrow } = E_{g} + 2\lambda_{c} + h = 1065$ meV, and it is the same for both P and AP configurations and does not vary with *U*, as seen in Fig. 5(c) and (d), as well as Tables 3 and 4.

For the P case, $E_{cK{\text{'}} \uparrow } = E_{g} - 2\lambda_{c} + U - \Delta \Omega = 635 + U$ meV, and increases with *U*, as seen in Fig. 5(c) and Table 3. So $E_{cK} = E_{cK \downarrow } = 1035 + U$ meV, $E_{cK{\text{'}} } = E_{cK{\text{'}} \uparrow } = 635 + U$ meV, $E_{c \uparrow } = E_{cK{\text{'}} \uparrow } = 635 + U$ meV, *E*_*c*↓_ = *E*_*cK’*↓_ = 1065 meV, as seen in Fig. 6(a) and (b) and Table 3. For the P case, *P*_*KK’*_ = -1 in the energy region [$635 + U$,$1035 + U$ meV], which is relatively wide and remains at 400 meV. As* U* increases, it moves parallel to the *E*_*F*_-axis as a whole, as seen in Fig. 6(c). *P*_*s*_ = 1 in the energy region [$635 + U$,1065 meV], which decreases with *U*, as seen in Fig. 6(d).

For the AP case, $E_{cK{\text{'}} \uparrow } = E_{g} - 2\lambda_{c} + h = 1035$ meV, and does not change with *U*, as seen in Fig. 5(d) and Table 4. So $E_{cK} = E_{cK \downarrow} = 1035 + U$ meV, *E*_*cK’*_ = *E*_*cK’*↑_ = 1035 meV, *E*_*c*↑_ = *E*_*cK’*↑_ = 1035 meV, *E*_*c*↓_ = *E*_*cK’*↓_ = 1065 meV, as seen in Fig. 6(e) and (f) and Table 4. For the AP case, *P*_*KK’*_ = -1 in the energy region [1035,$1035 + U$ meV], which increases with *U*, as seen in Fig. 6(g). As seen in Fig. 6(h), *P*_*s*_ = 1 in the energy region [1035,1065 meV], which is relatively narrow and remains at 30 meV as* U* increases.

Therefore, when 200 ≤ *U* < 400 meV, TMR = 1 in the energy region [$635 + U$,1035 meV], which decreases evidently with* U*, as shown in Fig. 9(c) and (d).

When* U* ≥ 400 meV, $U - \Delta \Omega \ge h$ holds. $E_{cK{\text{'}} \uparrow } = E_{g} - 2\lambda_{c} + U - \Delta \Omega = 635 + U$ meV, $E_{cK{\text{'}} \downarrow } = E_{g} + 2\lambda_{c} + U - \Delta \Omega = 665 + U$ meV, and they are the same for both P and AP configurations and increase with *U*, as seen in Fig. 5(c) and (d), as well as Tables 3 and 4. So $E_{cK} = E_{cK \downarrow } = 1035 + U$ meV, $E_{cK{\text{'}} } = E_{cK{\text{'}} \uparrow} = 635 + U$ meV, $E_{c \uparrow } = E_{cK{\text{'}} \uparrow } = 635 + U$ meV, $E_{c \downarrow } = E_{cK{\text{'}} \downarrow } = 665 + U$ meV, as seen in Fig. 6(a), (b), (e), and (f) and Tables 3 and 4. Thus *P*_*KK’*_ = -1 in the energy region [$635 + U$,$1035 + U$ meV], which is relatively wide and remains at 400 meV. As* U* increases, it moves parallel to the *E*_*F*_-axis as a whole, as shown in Fig. 6(c) and (g). In theory, *P*_*s*_ = 1 in the energy region [$635 + U$,$665 + U$ meV], which is relatively narrow and moves parallel to the *E*_*F*_-axis as* U* increases. However, when *U* = 450 meV, as seen in Fig. 6(b) and (f) and Tables 3 and 4, *E*_*c*↑_ (1096 meV) and *E*_*c*↓_ (1100 meV) are very close, making it difficult to see the energy region with *P*_*s*_ = 1 in Fig. 6(d) and (h).

Therefore, the energy region with TMR = 1 does not exist when* U* ≥ 400 meV, as described in Fig. 9(c) and (d).

**Fig. 7:**
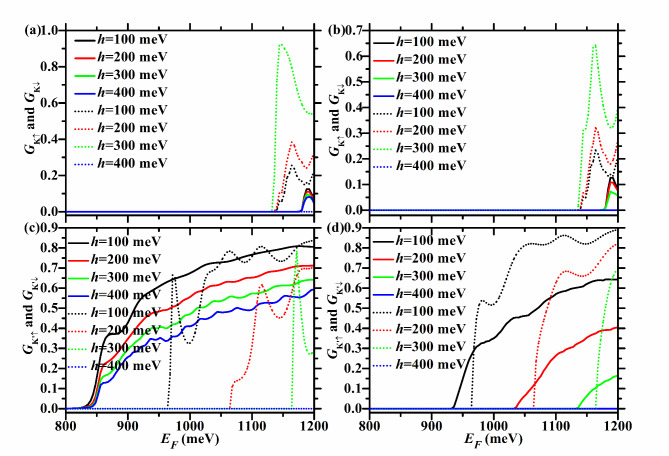
Spin- and valley-resolved conductance of the F/B/N/B/F WSe_2_ junction under different *h* and fixed *U* (100 meV) and ΔΩ (200 meV). The solid (dotted) lines correspond to spin-up (-down) electrons. In the left (right) column, the junction is in the P (AP) configuration.

**Fig. 8:**
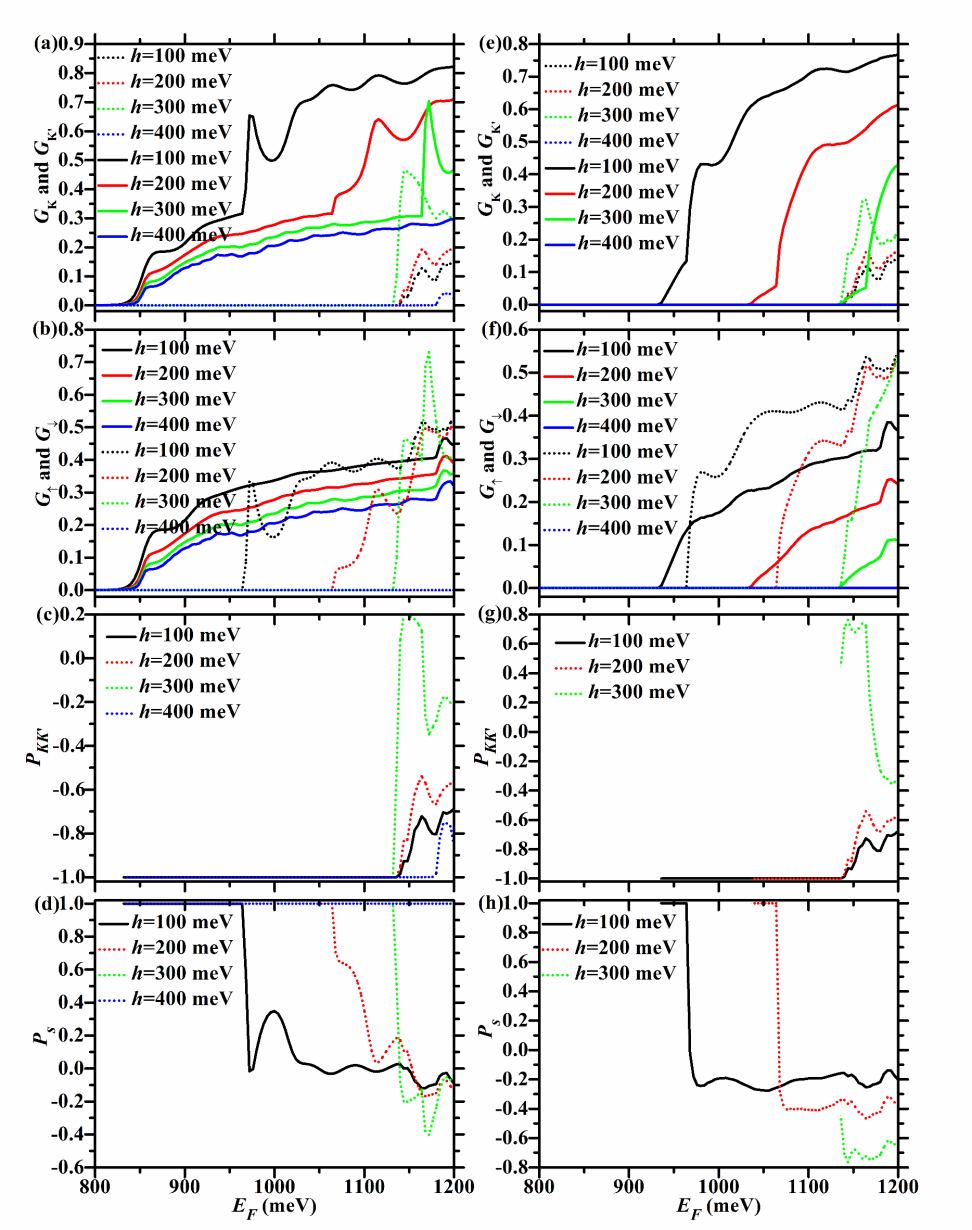
*G*_*K*_ (dotted lines),* G*_*K’*_ (solid lines), *G*_↑_ (solid lines), *G*_↓_ (dotted lines), *P*_*KK’*_, and *P*_*s*_ versus *E*_*F*_ for the F/B/N/B/F WSe_2_ junction with *U* = 100 meV and ΔΩ = 200 meV,* h* = 100, 200, 300, and 400 meV, respectively. In the left (right) column, the junction is in the P (AP) configuration.

**Fig. 9:**
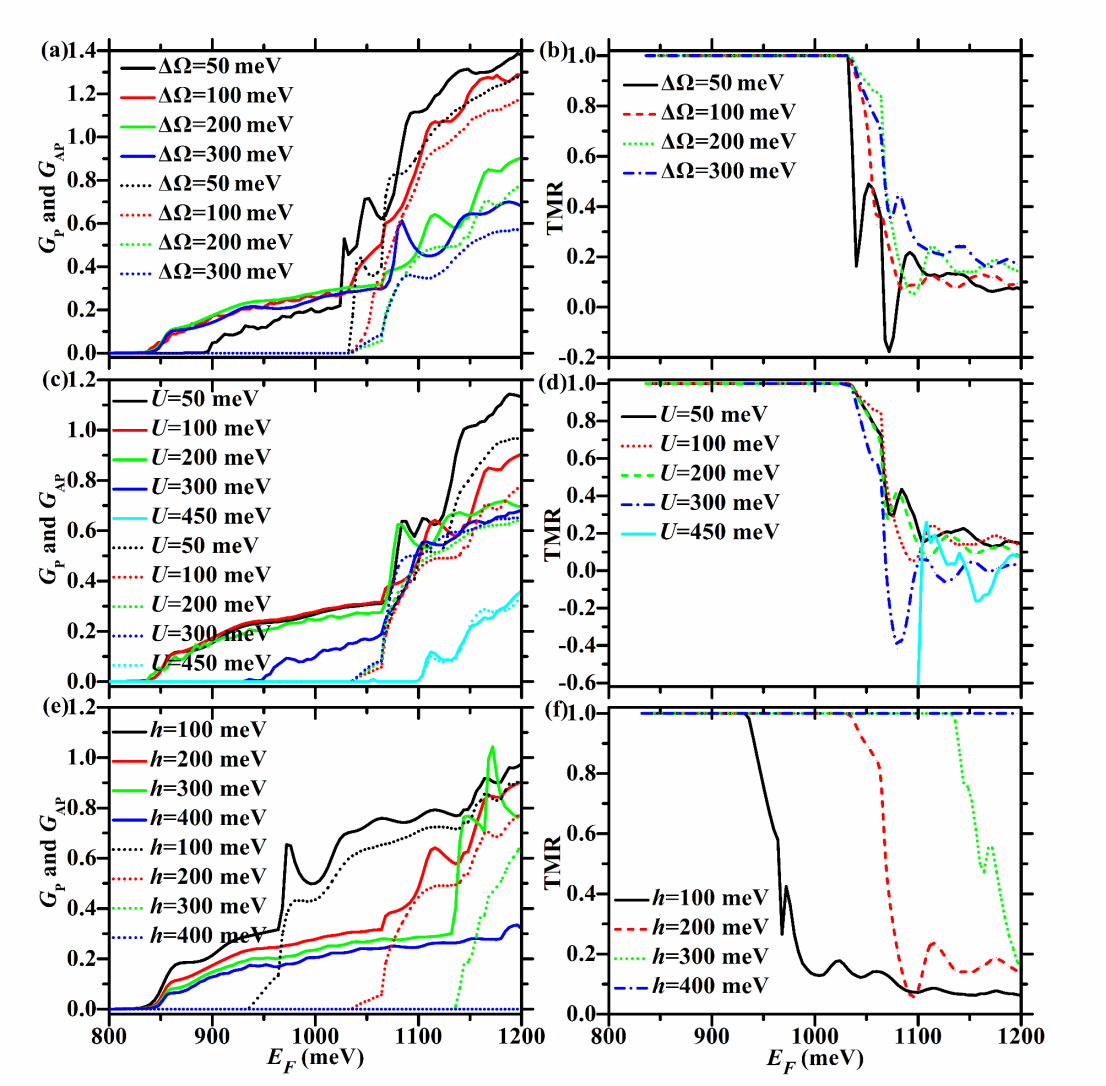
The total conductance (left column) of the F/B/N/B/F WSe_2_ junction in P and AP configurations, as well as the corresponding TMR (right column). The solid (dotted) lines correspond to the P (AP) configuration. Here *U* = 100 meV and *h* = 200 meV in the first row, *h* = 200 meV and ΔΩ = 200 meV in the second row, and *U* = 100 meV and ΔΩ = 200 meV in the third row.

Figures 7 and 8 show the spin- and valley-resolved conductance of the F/B/N/B/F WSe_2_ junction in P and AP configurations with *U* = 100 meV, ΔΩ = 200 meV, and different *h*. Figure 9 exhibits the corresponding total conductance as well as TMR, and Tables 5 and 6 depict the corresponding critical incident energy for the non-zero spin- and valley-resolved conductance.

**Table 5:** The critical incident energy (in units of meV) of non-zero spin- and valley-resolved conductance of the F/B/N/B/F WSe_2_ junction in the P configuration with *U* = 100 meV, ΔΩ = 200 meV, and *h* = 100, 200, 300, and 400 meV, respectively.

*h*	*E*_*cK*_$_\uparrow$	*E*_*cK*_$_\downarrow$	*E*_*cK’*_$_\uparrow$	*E*_*cK’*_$_\downarrow$	*E*_*cK*_	*E*_*cK’*_	*E*_*c*_$_\uparrow$	*E*_*c*_$_\downarrow$
100	1180	1140	812	968	1140	812	812	968
200	1180	1140	812	1068	1140	812	812	1068
300	1180	1140	808	1168	1136	808	808	1136
400	1180	> 1200	808	> 1200	1180	808	808	> 1200

**Table 6:** The critical incident energy (in units of meV) of non-zero spin- and valley-resolved conductance of the F/B/N/B/F WSe_2_ junction in the AP configuration with *U* = 100 meV, ΔΩ = 200 meV, and *h* = 100, 200, 300, and 400 meV, respectively.

*h*	*E*_*cK*_$_\uparrow$	*E*_*cK*_$_\downarrow$	*E*_*cK’*_$_\uparrow$	*E*_*cK’*_$_\downarrow$	*E*_*cK*_	*E*_*cK’*_	*E*_*c*_$_\uparrow$	*E*_*c*_$_\downarrow$
100	1180	1140	936	968	1140	936	936	968
200	1176	1136	1036	1068	1140	1036	1036	1068
300	1176	1136	1136	1168	1136	1136	1136	1136
400	> 1200	> 1200	> 1200	> 1200	> 1200	> 1200	> 1200	> 1200

For *U* = 100 meV and ΔΩ = 200 meV, $U - \Delta \Omega < 0 < h$ holds. So $E_{cK{\text{'}} \downarrow } = E_{g} + 2\lambda_{c} + h = 865 + h$ meV, and it is the same for both P and AP configurations and increases with *h*, as described in Fig. 7(c) and (d), as well as Tables 5 and 6. For the P case, $E_{cK \uparrow } = E_{g} + 2\lambda_{c} + U + \Delta \Omega = 1165$ meV, $E_{cK{\text{'}} \uparrow } = E_{g} - 2\lambda_{c} = 835$ meV, and they do not change with *h,* as seen in Fig. 7(a) and (c) and Table 5. For the AP case, $E_{cK{\text{'}} \uparrow } = E_{g} - 2\lambda_{c} + h = 835 + h$ meV, and increases with *h*, as shown in Fig. 7(d) and Table 6.

When* h* < 300 meV, $U + \Delta \Omega > h$ holds. $E_{cK \downarrow } = E_{g} - 2\lambda_{c} + U + \Delta \Omega = 1135$ meV and does not change with *h*, whether P or AP configuration, as depicted in Fig. 7(a) and (b), as well as Tables 5 and 6.

For the P case, *E*_*cK*_ = *E*_*cK*↓_ = 1135 meV, *E*_*cK’*_ = *E*_*cK’*↑_ = 835 meV, *E*_*c*↑_ = *E*_*cK’*↑_ = 835 meV, $E_{c\downarrow} = \min \{ 1135,865 + h\}$ meV, as seen in Fig. 7(a) and (b) and Table 5. Therefore, for the P case, as *h* increases, the energy region with *P*_*KK’*_ = -1 remains at [835,1135 meV], which is relatively wide and remains at 300 meV, as shown in Fig. 8(c). As shown in Fig. 8(d), *P*_*s*_ = 1 in the energy region [835,$\min \{ 865 + h,1135\}$ meV]. This region increases evidently with *h* when 0 < *h* < 270 meV, and remains at [835,1135 meV] when 270 ≤ *h* < 300 meV.

For the AP case, $E_{cK \uparrow } = E_{g} + 2\lambda_{c} + U + \Delta \Omega = 1165$ meV, and does not change with *h*, as shown in Fig. 7(b) and Table 6, just like in the P case. So *E*_*cK*_ = *E*_*cK*↓_ = 1135 meV, $E_{cK{\text{'}} } = E_{cK{\text{'}} \uparrow} = 835 + h$ meV, $E_{c \uparrow } = E_{cK{\text{'}} \uparrow } = E_{g} - 2\lambda_{c} + h = 835 + h$ meV, $E_{c \downarrow } = \min \{ 1135,865 + h\}$ meV, as seen in Fig. 8(e) and (f) and Table 6. For the AP case, *P*_*KK’*_ = -1 in the energy region [$835 + h$,1135 meV], which decreases evidently with *h*, as seen in Fig. 8(g). When *h* = 300 meV, the energy region with *P*_*KK’*_ = -1 disappears completely. As shown in Fig. 8(h), *P*_*s*_ = 1 in the energy region [$835 + h$,$\min \{ 865 + h,1135\}$ meV]. When 0 < *h* < 270 meV, this region remains at [$835 + h$,$865 + h$ meV], with a width of 30 meV. When 270 < *h* < 300 meV, it decreases evidently with *h*. When *h* = 300 meV, it disappears completely.

Therefore, when *h* < 300 meV, TMR = 1 in the energy region [835,$835 + h$ meV], which is relatively wide and increases rapidly with* h*, as shown in Fig. 9(e) and (f).

When* h* ≥ 300 meV, $U + \Delta \Omega \le h$ holds. So $E_{cK \downarrow } = E_{g} - 2\lambda_{c} + h = 835 + h$ meV, and increases with *h*, regardless of P or AP configuration, as seen in Fig. 7(a) and (b), as well as Tables 5 and 6.

For the P case, $E_{cK} = \min \{ 1165,835 + h\}$ meV, *E*_*cK’*_ = *E*_*cK’*↑_ = 835 meV, *E*_*c*↑_ = *E*_*cK’*↑_ = 835 meV, $E_{c \downarrow} = E_{cK \downarrow} = 835 + h$ meV, as seen in Fig. 8(a) and (b) and Table 5. For the P case, *P*_*KK’*_ = -1 in the energy region [$835$,$\min \{ 835 + h,1165\}$ meV], as shown in Fig. 8(c). When 300 < *h* < 330 meV, it increases with *h*. When *h* ≥ 330 meV, it remains at [835,1165 meV], with a width of 330 meV. *P*_*s*_ = 1 in the energy region [835,$835 + h$ meV], which increases with *h*, as seen in Fig. 8(d)*.*

For the AP case, $E_{cK \uparrow } = E_{g} + 2\lambda_{c} + h = 865 + h$ meV, and increases with *h*, as seen in Fig. 7(b) and Table 6. So $E_{cK} = E_{cK \downarrow } = 835 + h$ meV, $E_{cK{\text{'}} } = E_{cK{\text{'}} \uparrow } = 835 + h$ meV, $E_{c \uparrow } = E_{cK{\text{'}} \uparrow } = 835 + h$ meV, $E_{c \downarrow } = E_{cK \downarrow } = 835 + h$ meV, as seen in Fig. 8(e) and (f) and Table 6. For the AP case, the energy region with* P*_*KK’*_ = -1 (*P*_*s*_ = 1) does not exist, as seen in Fig. 8(g), (h).

Therefore, when *h* ≥ 300 meV, TMR = 1 in the energy region [835,$835 + h$ meV], which increases with *h*, as seen in Fig. 9(e) and (f).

## Conclusion

In conclusion, by tuning the exchange field* h* in the FM region, the electrostatic potential *U* and CPL intensity ΔΩ in the barrier region, the energy region of full spin and valley polarizations as well as large TMR in the F/B/N/B/F WSe_2_ junction can be modulated, and the underlying physical mechanisms have been unveiled. We have derived the minimum incident energy of non-zero spin- and valley-resolved conductance, which is demonstrated by numerical calculations. The sign of the valley polarization *P*_*KK’*_ depends on the helicity of the CPL, which does not happen to TMR and* P*_*s*_. The energy region with TMR = 1 increases with *h* rapidly, remains unchanged first and then decreases as *U* increases, and has little dependence on ΔΩ. The energy region with *P*_*KK’*_ = -1 or *P*_*s*_ = 1 for the P case is much wider than that of the AP case, and they both increase evidently with ΔΩ. With increasing *h*, the *P*_*s*_ = 1 plateau widens for the P configuration, while the *P*_*KK’*_ = -1 or *P*_*s*_ = 1 plateau narrows for the AP configuration. For the P configuration, as *U* increases, the energy region with *P*_*KK’*_ = -1 (*P*_*s*_ = 1) increases (decreases) and that of *P*_*s*_ = 1 (*P*_*KK’*_ = -1) remains unchanged when *U* is relatively small (large). When *U* increases to a certain degree, the plateaus with *P*_*KK’*_ = -1 or *P*_*s*_ = 1 move parallel to the *E*_*F*_-axis, regardless of P or AP configuration. Our research helps the practical application of ferromagnetic WSe_2_ double-barrier junctions in fabricating spin-valleytronic and TMR devices.

## Data Availability

Data will be made available on request.
